# Effects of surfactants on luminescence intensity and duration of the minimal luciferase picALuc

**DOI:** 10.2142/biophysico.bppb-v23.0023

**Published:** 2026-07-11

**Authors:** Yuki Ohmuro-Matsuyama, Kento Motoyama, Genta Kamiya, Ryogo Takai, Nobuo Kitada, Mitsuru Hattori, Shojiro A. Maki, Takeharu Nagai, Tadaomi Furuta

**Affiliations:** 1 Technology Research Laboratory, Shimadzu Corporation, Soraku-gun, Kyoto 619-0237, Japan; 2 Graduate School of Informatics and Engineering, The University of Electro-Communications, Chofu, Tokyo 182-8585, Japan; 3 SANKEN, The University of Osaka, Ibaraki, Osaka 567-0047, Japan; 4 School of Life Science and Technology, Institute of Science Tokyo, Yokohama, Kanagawa 226-8501, Japan

**Keywords:** bioluminescence, surfactant, luminescence intensity, luminescence lifetime

## Abstract

Bioluminescence is an oxidation-mediated chemical reaction involving the luciferase enzyme and the luciferin substrate. To date, various artificial luciferases have been developed, and their luminescence properties have been investigated. However, very few studies have focused on the solvent environment in which these luminescent reactions occur, and the details of their influence on luminescent phenomena remain unclear. In this study, we investigated the effects of various surfactants on the activity of the luciferase picALuc. To this end, we analyzed the effects of adding diverse surfactants on the luminescence properties of the luciferase picALuc. The results showed that the addition of a surfactant enhanced the luminescence intensity and extended the duration of luminescence. Dynamic light scattering measurements and microscopic observations revealed that micelle-like particles were formed by the surfactant, which suppressed luminescence inhibition. This study provides our original method for adding surfactants to enzymatic reactions, such as those involving hydrophobic substrates.

## Significance

Bioluminescence by luciferin–luciferase reactions is influenced by the solvent environment, yet this aspect has been little explored. This study examined how surfactants affect the luciferase picALuc and found that they increased luminescence intensity and duration. DLS and microscopy observations showed that surfactants formed micelle-like particles that localized luminescence and fluorescence from coelenterazine metabolites. Although these metabolites inhibited luminescence, surfactants alleviated this inhibition. These results suggest that micelles alter metabolite distribution and enhance luminescence, offering a strategy for enzymatic reactions involving hydrophobic substrates.

## Introduction

Luminescent organisms live in the dark by emitting light through oxidation and utilizing enzymatic reactions [1]. To date, at least 11 types of luciferin have been identified, including D-luciferin in fireflies, vargulin (*Cypridina* luciferin) in sea-fireflies, and coelenterazine (CTZ) in copepods, which is also used in the photoprotein of *Aequora* jellyfish. Research into their biosynthesis is ongoing [2], and interestingly, it has been suggested that CTZ and vargulin are formed by the cyclization reactions of three amino acids: F-Y-Y and R-I-W, respectively [3,4]. In addition, the structures and mechanisms of the enzymes that catalyze these substrates, namely luciferases, have recently been elucidated, including firefly luciferase FLuc [5], *Renilla* luciferase RLuc [6], *Gaussia* luciferase GLuc [7], and the developed luciferase NanoLuc derived from deep-sea shrimp [8]. A common feature is that they all emit light through the so-called luciferin–luciferase (L-L) reaction that uses dioxetane as a reaction intermediate, but that there are some exceptions, such as the bacterial bioluminescent system, that uses hydroperoxide [9]. In most L-L reactions, oxygen (O_2_) is taken in through oxidation, and carbon dioxide (CO_2_) is released; therefore, the luminescent reaction may be considered a type of molecular (enzymatic) respiration, in which excess energy is released as light. It is unknown how such a system formed during evolutionary history; however, it may be related to the elimination of oxidative stress [10].

In recent years, artificial luciferases have been intensively investigated because of their utility, and in addition to NanoLuc (19 kDa), TurboLuc, ALuc, and LuxSit have been developed. TurboLuc (16 kDa) was developed from the luciferase of *Metridia pacifica* [11], and ALuc (~21 kDa) and its variants have been developed based on the sequence information of multiple copepod luciferases [12–14]. In contrast, LuxSit (13.9 kDa) was developed using deep learning based on non-luciferase enzymes [15]. Recently, we developed a miniaturized small luciferase, picALuc (13 kDa), by removing the N- and C-terminal residues from ALuc [16]. Furthermore, we recently made improvements to picALuc, by enhancing its luminescence through addition of a charged oligopeptide (picALuc2.0 [17]) and using mutational analyses to convert it to a glow type (picA-Re [18]). In addition to these modifications of the enzyme, attempts have also been made to modify the substrate. A substrate modified at the C-6 position of CTZ produced fairly dim luminescence at a wavelength of 637 nm, and a CTZ analog fused with the dye cyanine 5 (Cy5) at the same position emitted light at a near-infrared wavelength of 700 nm through unique through-bond energy transfer [19]. This study aimed to determine whether changes in the solvent environment affect luminescence, apart from changes in the solutes such as enzymes and substrates mentioned above.

Now, if we look at the way humans live today, they live by breathing, obtaining nutrients from food, and excreting waste [20,21]. Surfactants with both hydrophilic and hydrophobic groups are used as key ingredients in soaps and detergents [22]. Protein (enzyme) function is regulated by allosteric effects or additives [23,24]. This study began with the question, does adding sugar or a surfactant to the above luminescent enzyme reaction change enzyme activity? Unfortunately, preliminary investigation did not show any significant effects of addition of sugars on the luminescent enzyme reaction (data not shown). However, when a surfactant was added, slightly brighter light was emitted in the luminescent reaction. Moreover, when the tube was gently shaken, it was observed that bubbles appeared at the water surface. To date, a detailed analysis of the addition of various surfactants to the reactions of luminescent enzymes has not been performed, and the mechanisms underlying their luminescence remain unknown.

Here, as an extension of our previous development of the minimal luciferase picALuc, we analyzed the changes in luminescence when various surfactants were added to the enzymatic reaction of picALuc (1.0) [16] with the substrate coelenterazine *h* (CTZ*h*). With the addition of surfactants, different concentration-dependent behaviors appeared for both the luminescence intensity and duration of luminescence. Furthermore, it is suggested that the reaction products (substrate metabolites) are involved in these phenomena.

## Materials and methods

### Reagents

Nonylphenoxypolyethoxylethanol (NP-40) was purchased from FUJIFILM Wako Pure Chemical Corporation (Osaka, Japan). Polyoxyethylene sorbitan monolaurate (Tween 20) and 4-(1,1,3,3-tetramethylbutyl)phenyl-polyethylene glycol (Triton X-100) were from Thermo Fisher Scientific (MI, USA). A Detergent Screening Set containing 3-[(3-Cholamidopropyl)dimethylammonio]propanesulfonate (CHAPS), *n*-Octyl-β-D-thioglucoside, and *n*-Octanoyl-*N*-methyl-D-glucamine (MEGA-8) was from Dojindo, (Kumamoto, Japan). For the synthesis of 3-benzyl-5-(4-hydroxyphenyl) pyrazin-2(1*H*)-one (CTO), coelenteramine (CTM), and coelenteramide (CTMD; oxyluciferin), the starting materials, reagents, and solvents were purchased from Tokyo Chemical Industry Co., Ltd. (Tokyo, Japan), FUJIFILM Wako Pure Chemical, Kanto Chemical Co., Inc. (Tokyo, Japan), and Sigma–Aldrich (Tokyo, Japan), respectively, and were used without further purification. All other reagents were of special grade (FUJIFILM Wako Pure Chemical).

### Protein expression and purification

*Escherichia coli* SHuffle T7 lysY cells (New England Biolabs (MA, USA)) were transformed with picALuc1.0, cultured, and induced for protein expression overnight at 16°C in a 100-mL culture containing 0.4 mM isopropyl-β-D-thiogalactopyranoside. Proteins from the cytoplasm of cells expressing picALuc were purified using a His-tag in the pET32 vector by immobilized metal affinity chromatography using TALON resin (Takara Bio, Shiga, Japan) according to the manufacturer’s protocol, including imidazole-containing wash and elution steps [16]. The concentrations of purified luciferases were determined via electrophoresis using a Mini-PROTEAN-TGX Stain-Free gel co-loaded with various concentrations of bovine serum albumin (BSA) as the concentration standard and calculated using the GelDoc system (Bio-Rad, CA, USA). The protein mixed with 15% glycerol and stored at 4 or –80°C before use.

### Measurement of luminescence intensity

The enzyme and substrate were diluted in buffer (137 mM NaCl, 9.6 mM KH_2_PO_4_, 2.7 mM KCl, pH 4.32). The enzyme solution with or without each surfactant (20 μL each) was dispensed to a 96-well half-area plate (3694, Corning–Coastar, NY, USA). The luminescence intensity was measured after injection of 20 μL of 2×substrate solution using a GloMax Navigator Microplate Luminometer (Promega, WI, USA). In the analysis of the luminescence lifetime, the luminescence intensity was measured at 2 min intervals. The luminescence half-lives were estimated using exponential curve fitting. In cases where the data did not fit the usual exponential decay, the half-lives were calculated by interpolation as the time when the luminescence intensity reached half of the maximum intensity.

### Simplified enzyme-linked immunosorbent assay detection system

In a typical enzyme-linked immunosorbent assay (ELISA) system, a target-binding protein is immobilized on a basal plate, and a sample, potentially containing the target, is added for measurement. To detect the target bound to the target-binding protein on the basal plate, a procedure similar to a sandwich ELISA was used. A biotinylated target-binding protein was added, followed by addition of a streptavidin-linked enzyme for quantification of the target [25] (Figure S1). In this study, we constructed a simplified system using biotinylated BSA as the target-binding protein and the streptavidin-fused enzyme luciferase picALuc as the target-containing sample.

The gene encoding streptavidin was synthesized by Eurofin Genomics (Tokyo, Japan) and inserted into a plasmid containing picALuc [16]. Streptavidin-fused picALuc was expressed and purified as described previously. BSA was biotinylated using the Biotin Labeling Kit-NH2 (Cosmobio, Tokyo, Japan) according to the manufacturer’s protocol. Nonbiotinylated- or biotinylated BSA was diluted (10 μg/mL) in phosphate-buffered saline (PBS), and dispensed to 96-well plate (3912, Corning–Coastar) and incubated at 4°C overnight, and then removed. The wells were washed with PBST (0.1% Tween) and mixed with 20% ImmunoBlock (KAC, Kyoto, Japan) in PBS, and incubated at room temperature for 1 hour. After removing the blocking reagent and washing, streptavidin-fused picALuc diluted into 5% Immunoblock in PBST (1 μg/mL) was added and incubated at room temperature for 1 h. After removing streptavidin-fused picALuc and washing, 100 μL of PBS was added. The luminescence intensity was measured immediately after adding 240 μM of CTZ*h* (100 μL) and 0–0.10% NP-40 using a GloMax Navigator Microplate Luminometer was added.

### Dynamic light scattering measurement

NP-40 (0.01%), picALuc (10 nM), CTZ*h* (2.5 μM), and EtOH (0.1%) were dissolved with the buffer (137 mM NaCl, 9.6 mM KH_2_PO_4_, 2.7 mM KCl, pH 4.32). The mixtures (100 μL) were dispensed to UV-Cuvette micro (Merck, NJ, USA), and the particle size distribution was measured using an ELSZ-2000 particle size analyzer (Otsuka Electronics, Hiroshima, Japan).

### Syntheses of CTO, CTM, and CTMD

Silica gel 70 F254 thin-layer chromatography (TLC) plates (FUJIFILM Wako Pure Chemical) were used for analytical TLC, and silica gel 60 N (spherical, neutral, Kanto Chemical, Tokyo, Japan) was used for column chromatography. 1H and 13C NMR spectra were obtained using a JEOL ECA-500 or ECZL-500R instrument (500 MHz for ^1^H and 126 MHz for ^13^C) (JEOL, Tokyo, Japan). The molecular weights were determined using matrix-assisted laser desorption/ionization mass spectrometry (JMS-S3000 SpiralTOF^TM^-plus 2.0) (JEOL). HPLC analyses were performed on a Thermo Scientific LCQ Fleet system equipped with a Thermo Scientific Hypersil GOLD column (5 μm, 150×4.6 mm).

CTM, CTO, and CTMD were synthesized according to the following scheme. Compound 2a was synthesized by Negishi cross-coupling of commercially available 2-amino-3,5-dibromoaminopyrazine 1a with commercially available zinc chloride, benzylmagnesium chloride, and bis (triphenylphosphine)palladium(II) dichloride. CTM was synthesized by the Suzuki-Miyaura cross-coupling of compound 2a with commercially available 4-hydroxyphenyl boronic acid and tetrakistriphenylphosphine palladium(0). Compound 3a was synthesized following the same procedure as that used for the synthesis of 4-methoxyphenylboronic acid. CTO was synthesized by mixing CTM and sodium nitrite in sulfuric acid. Compound 4a was amidated with commercially available 4-methoxyphenylacetyl chloride to obtain compound 5a. Finally, 5a was demethylated with boron tribromide to obtain CTMD (Scheme S1 and Method S1).

### Microscopic observation

Native CTZ (nCTZ) dissolved in EtOH was suspended with NP-40 (0.01%) in PBS to a final concentration of 30 μM. A final concentration of 10 nM picALuc was used for observation. The fluorescence and bioluminescence of nCTZ were observed using an inverted fluorescence microscope (IX83, Olympus) equipped with a ×60 objective (PlanApo N, Olympus) and an EM-CCD (iXon Ultra888, Andor Technology). Fluorescence images were acquired using a filter cube (U-FBNA; Olympus). The exposure time for bioluminescence images was 30 s. CTO (0.5 μM), CTM (50 nM), and CTMD (0.5 μM) dissolved in methanol (MeOH), and C-terminal mCherry-fused picALuc (GS-linker) (57 nM) [16] were suspended in NP-40 (0.01%) in PBS; the final concentration of MeOH was 0.1%. The particles and fluorescence of CTZ, CTO, CTM, CTMD, and mCherry were observed using a BZ-X810 fluorescence microscope with a 40× lens (Keyence, Osaka, Japan).

### Sonication

To disperse particle aggregates, sonication was performed for 3 min using a Spin Dryer Lite VC-36R (TAITEC, Nagoya, Japan).

### Liquid chromatography–mass spectroscopy Measurement

nCTZ (40 μM) dissolved in MeOH was reacted with picALuc (50 nM) in PBS. The reaction was performed at room temperature for either 10 or 60 min. To stop the reaction, hydrochloric acid diluted 100-fold (1/10 volume of the reaction solution) was added. The enzymes were removed by adding five times the volume of MeOH to the reaction solution, followed by centrifugation (20,000×g for 15 min). The supernatant was collected for liquid chromatography–mass spectroscopy (LC–MS) analysis.

MS analysis was performed using a triple quadrupole mass spectrometer (LCMS-8060 system (Shimadzu Corporation, Japan) operating in the multiple reaction monitoring mode. Detection was performed using an electrospray ionization source in both positive and negative ion modes. The interface temperature was maintained at 300°C, with a heating gas flow rate of 10 L/min, a nebulizer gas flow rate of 3 L/min, and a drying gas flow rate of 10 L/min. The desolvation line temperature was set to 250°C.

Chromatographic separation was performed using the Nexera XS Inert UHPLC system (Shimadzu Corporation, Kyoto, Japan). Separation was performed using a Wakosil 5C4 column (2.0 mm i.d.×250 mm; Wako). The mobile phases used were water (mobile phase A) and acetonitrile (mobile phase B) containing 0.1% formic acid. The flow rate was set to 0.2 mL/min. The gradient elution program began with 30% B, increased to 45% B over 15 min, maintained at 99% B for 3 min, and finally equilibrated at 0% B for 7 min. The column temperature was maintained at 25°C, and the injection volume was 1 μL.

For the semi-quantitative analysis of the enzymatic reaction products, an external standard method was employed. Four standard compounds associated with the picALuc enzymatic reaction (nCTZ, CTO, CTM, and CTMD) were dissolved in 50% MeOH and mixed to ensure equal concentrations. Standard mixtures of these compounds were prepared at six concentration levels: 25 μM, 12.5 μM, 5 μM, 0.5 μM, 0.05 μM, and 0.005 μM. Mass spectrometry data were analyzed using the LabSolutions software (Shimadzu Corporation). Compound peak identification and quantification of the enzymatic reaction samples were performed by referencing the MS/MS data of the standard compounds.

During the luminescence measurement of nCTZ, the signals differed in the presence and absence of NP-40 even at 0 min (no enzyme added). This discrepancy was likely due to the ionic effect of NP-40, which caused overestimation of nCTZ. Therefore, the amount of nCTZ measured with NP-40 was normalized to that measured without NP-40.

## Results

### Changes in luminescence patterns due to the addition of surfactants

**Figure 1:**
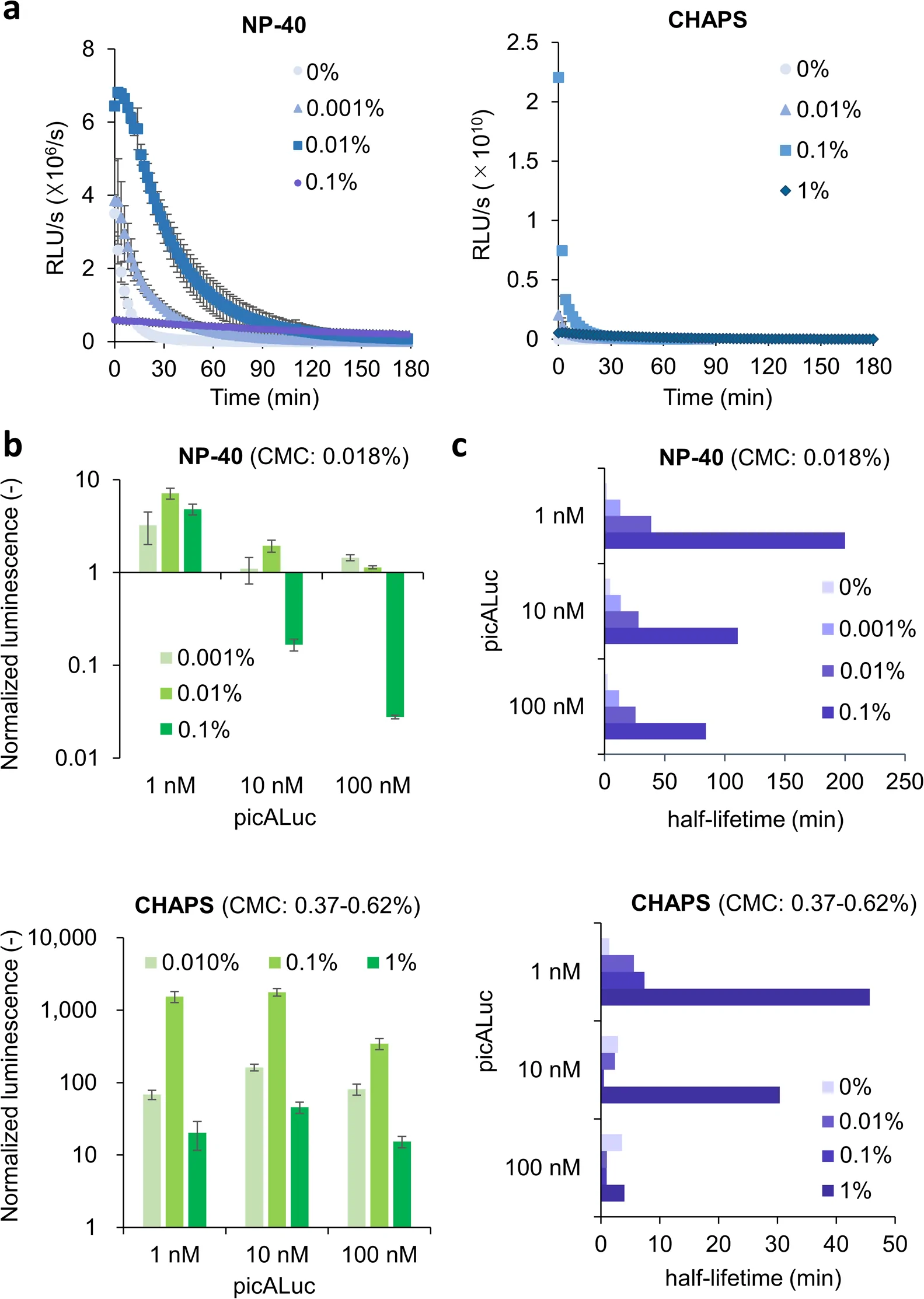
Changes of luminescence patterns of picALuc by adding surfactants. (a) Luminescence duration with NP-40 (0, 0.001, and 0.01%) and CHAPS (0, 0.01, 0.1, and 1%). picALuc: 10 nM and CTZ*h*: 2.5 μM. (b) Effects of the surfactants on the maximum luminescence intensities. (c) Those on half-life times. Plots represent mean±SD (n=3) in (a) and (b), and half-life times in (c) were estimated from the mean luminescence curves.

**Figure 2:**
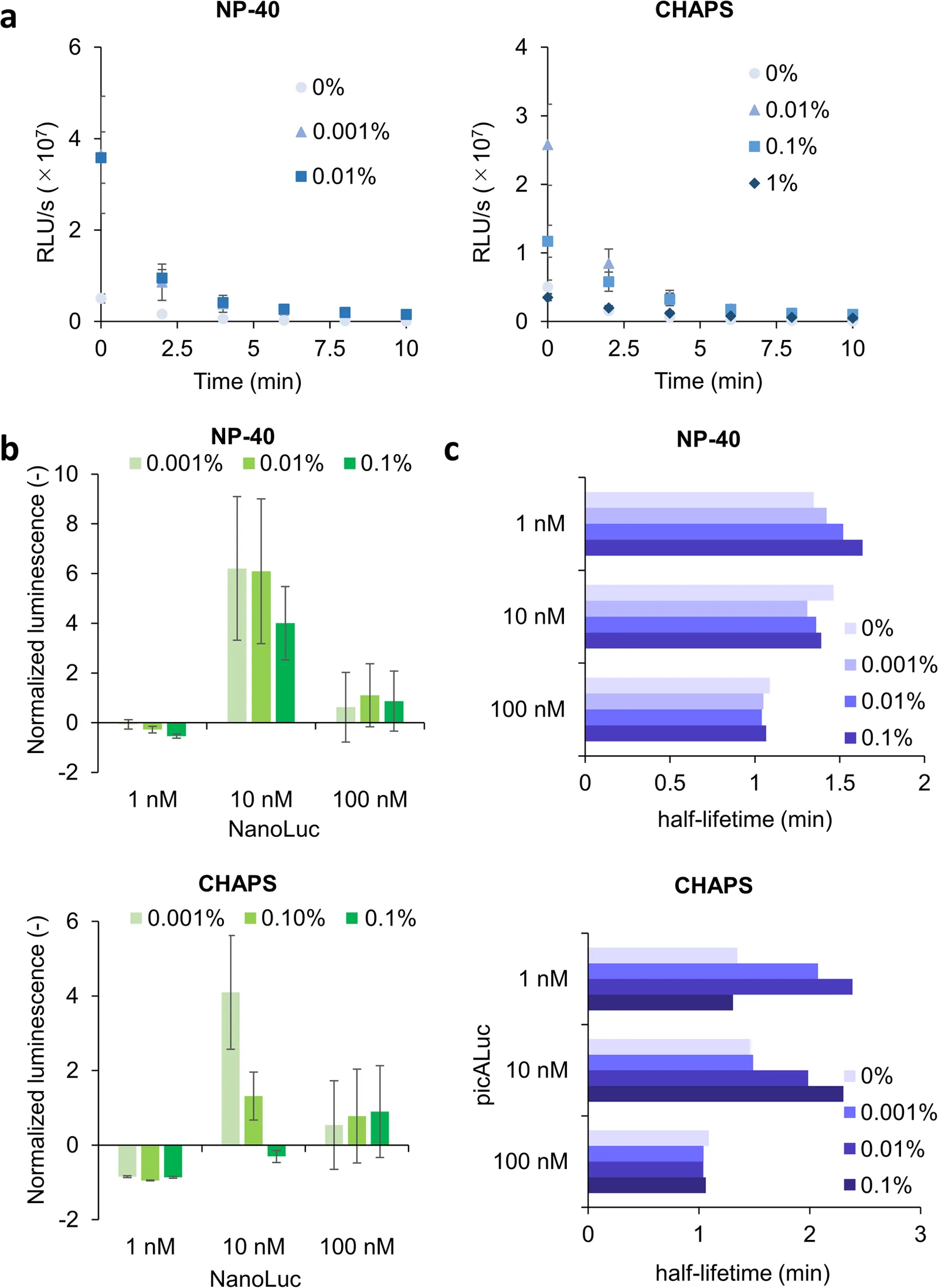
Changes of luminescence patterns of NanoLuc by adding surfactants. (a) Luminescence duration with NP-40 (0, 0.001, and 0.01%) and CHAPS (0, 0.01, 0.1, and 1%). NanoLuc: 1 nM and CTZ*h*: 2.5 μM. (b) Effects of the surfactants on the maximum intensities. (c) Those on half-life times. Plots represent mean±SD (n=3) in (a) and (b), and half-life times in (c) were estimated from the mean luminescence curves.

To prepare the enzyme solutions with various surfactants, picALuc was mixed with a NP-40, CHAPS, Tween 20, Triton X-100, *n*-Octyl-β-D-thioglucoside or MEGA-8 over a wide range of critical micelle concentrations (CMCs) ranging from 0.0037 to 4.14%. The substrate CTZ*h* was dissolved in ethanol (EtOH) and diluted with the reaction buffer (substrate solution). To examine the effects of the surfactants, luminescence intensities were measured after mixing equal volumes of the enzyme and substrate solutions. It was found that the addition of surfactants changed the intensity and decay (or duration) of the luminescence. These changes differed for each surfactant and depended on the concentrations of both surfactant and picALuc (Figures 1 and S2). The surfactants had significant effects on intensity enhancement (Figures 1b and S2a), particularly at low enzyme concentrations. Moreover, higher surfactant concentrations increased the luminescence duration, although the addition of excess surfactants led to a decrease in the intensity in some cases (Figures 1c and S2b). Among the surfactants tested in this study, *n*-Octyl-β-D-thioglucoside was the least effective. In contrast, CHAPS significantly enhanced the intensity (up to about 1,000-fold at 1 and 10 nM), and the effect of NP-40 on the luminescence duration was the most significant (up to about 200-fold at 1 nM). Moreover, to verify the effects of surfactants on other enzymes, we performed similar experiments using NanoLuc, a representative luciferase that reacts with CTZ and CTZ analogues [26]. Although the effects under the present conditions were smaller than those of picALuc, changes in intensity and duration of about 6- and 2-fold, respectively, occurred (Figure 2). These results suggested that the addition of surfactants can have at least weak, but similar effects on other enzymes.

### Application of surfactant addition effect to detection system

**Figure 3:**
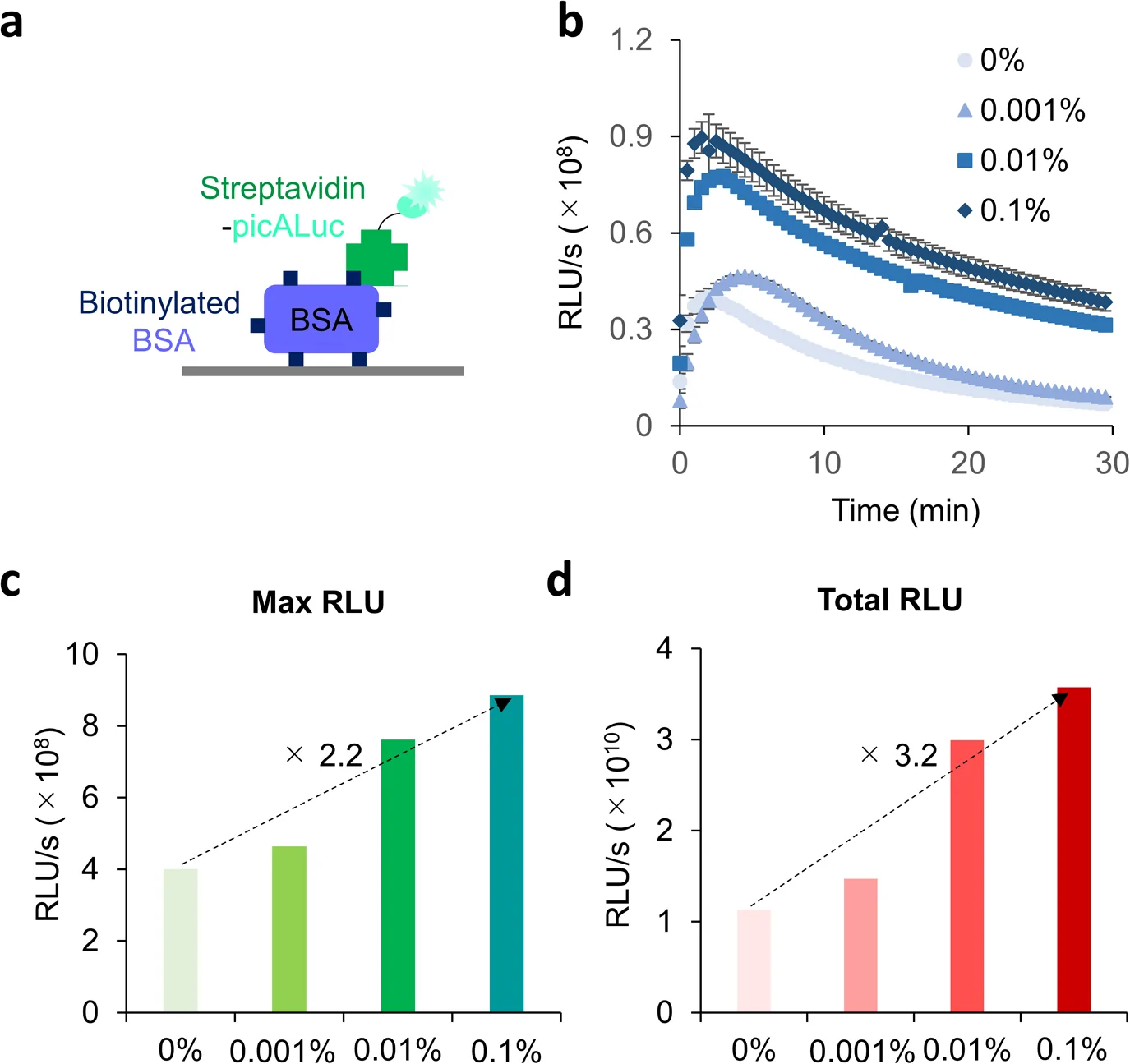
Demonstration adding surfactant to the detection system. (a) Schematic diagram of the mock detection system. (b) Luminescence duration with NP-40 (0, 0.001, 0.01, and 0.1%). (c) The average values of the maximum intensities. (d) The average values of the total intensities over 30 min.

We investigated whether the effect of surfactant addition can be applied to a target detection system such as an enzyme-linked immunosorbent assay (ELISA) [27]. We used a simplified ELISA detection system with biotinylated BSA and streptavidin-fused picALuc, and examined whether the addition of a surfactant could change the intensity and duration of the signal (Figure 3a). The results showed a concentration-dependent effect of up to 0.1% NP-40 on the intensity and duration of luminescence (Figure 3b). The maximum intensity (Max RLU) with 0.1% NP-40 was 2.2 times higher than that without NP-40 (Figure 3c), and the total luminescence intensity (Total RLU) integrated over 30 min after the addition of CTZ*h* was 3.2 times higher than that without NP-40 (Figure 3d). Further optimization of surfactant utilization will expand the dynamic range of luciferase-based detection systems and facilitate measurements.

In the above sections, we have shown that surfactants can alter the intensity and duration of luminescence, and that this effect can be applied to detection systems. In the following sections, we examine the phenomena that occur in solution and their relationships with the chemical species involved in the reaction.

### Micelle-like particle formation in luminescent reaction solution containing surfactants

**Figure 4:**
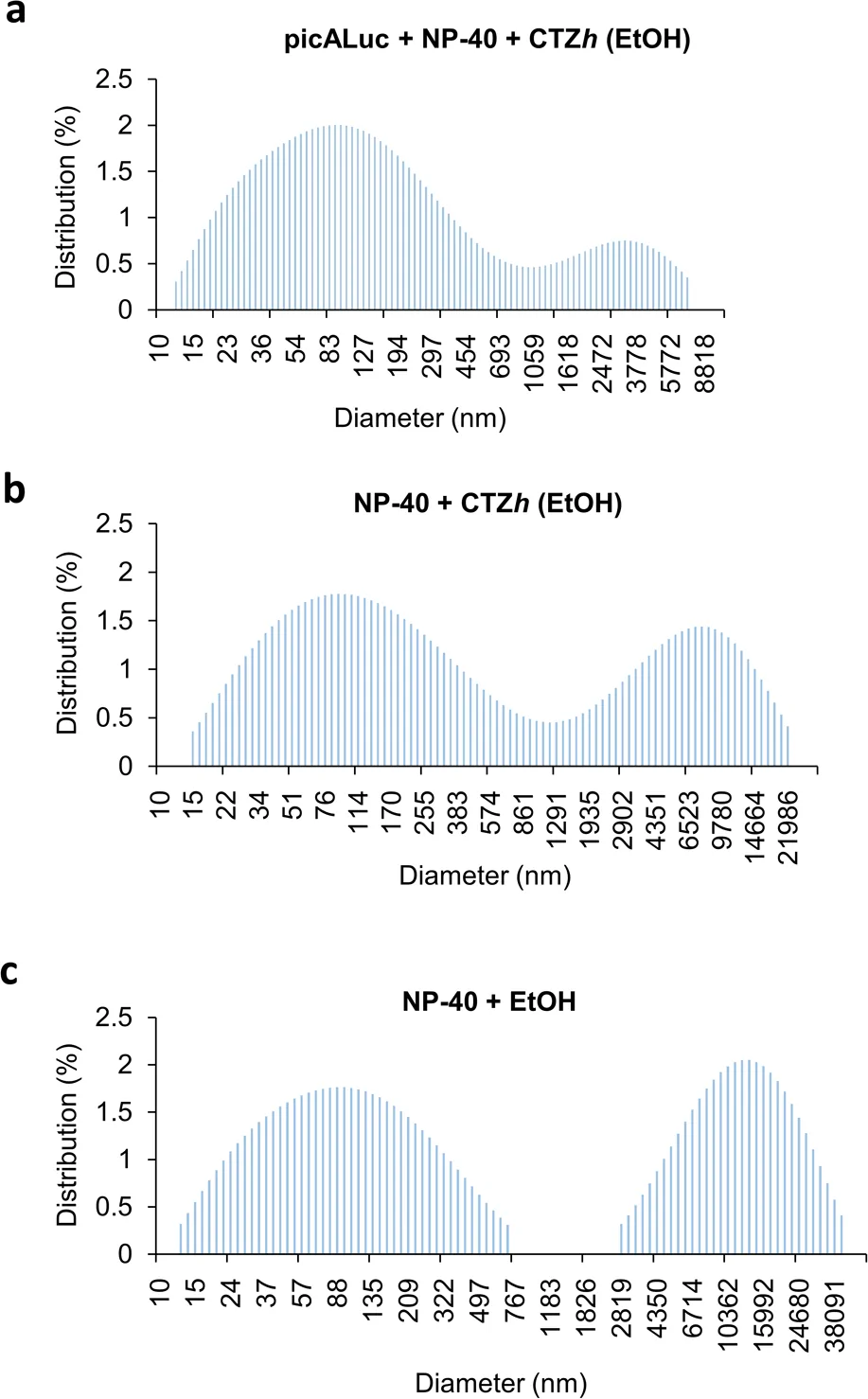
Particle formation by the mix of surfactant and EtOH. (a) picALuc+NP-40+CTZ*h* (EtOH). (b) NP-40+CTZ*h* (EtOH). (c) NP-40+EtOH. In each, picALuc (10 nM), NP-40 (0.01%), CTZ*h* (2.5 μM), and EtOH (0.1%).

From the above results (Figures 1 and S2), we noticed that surfactants with higher CMC required higher concentrations of substrates for sustained luminescence. For 10 nM picALuc, the luminescence with low CMC surfactants (0.0037–0.018%): Tween 20, Triton X-100, and NP-40 was sustained at concentrations of 0.001–0.1%, whereas that with high CMC surfactants (CMC: 0.37–4.14%) such as CHAPS, *n*-octyl-β-D-thioglucoside, and MEGA-8 required concentrations of at least 1%. These facts led us to believe that micelles or other micellar particles may be formed in reaction systems containing these surfactants. Therefore, we performed dynamic light scattering (DLS) measurements. Two peaks (approximately 100 and 4,000 nm) were observed in a mixture of picALuc, NP-40, and CTZ*h* dissolved in EtOH (Figure 4a). The distributions showed a wide range of particular sizes, and the distribution range changed over time after mixing. To determine the factors contributing to micelle-like particle formation, enzyme and substrate components were sequentially removed from the mixture. When picALuc was removed from this system, similar particles were observed, indicating that picALuc was not involved in the formation of micelle-like particles (Figure 4b). When CTZ*h* was removed from the system, that is, NP-40 was diluted with the buffer, no signal was observed, whereas when NP-40 (0.01%) was mixed with EtOH, a distribution of particles (re-)appeared (Figure 4c). These results demonstrate that the EtOH used to dissolve the substrate causes the formation of micelles or micelle-like particles by surfactants, even at surfactant concentrations much lower than the CMC.

Since substrates are often dissolved in MeOH rather than in EtOH, we examined whether MeOH would have a similar effect. After dissolving NP-40 in MeOH, a luminescence pattern was observed in the presence of 0.001–0.1% NP-40. Similar to when NP-40 was diluted with EtOH, a concentration-dependent increase in luminescence intensity and duration was observed (Figure S3), indicating that micelle-like particles were formed by the combination of the surfactant and alcohol used for substrate dissolution.

### Colocalization of luminescence signal, luciferase, substrate, and substrate metabolites on micelle-like particles

**Figure 5:**
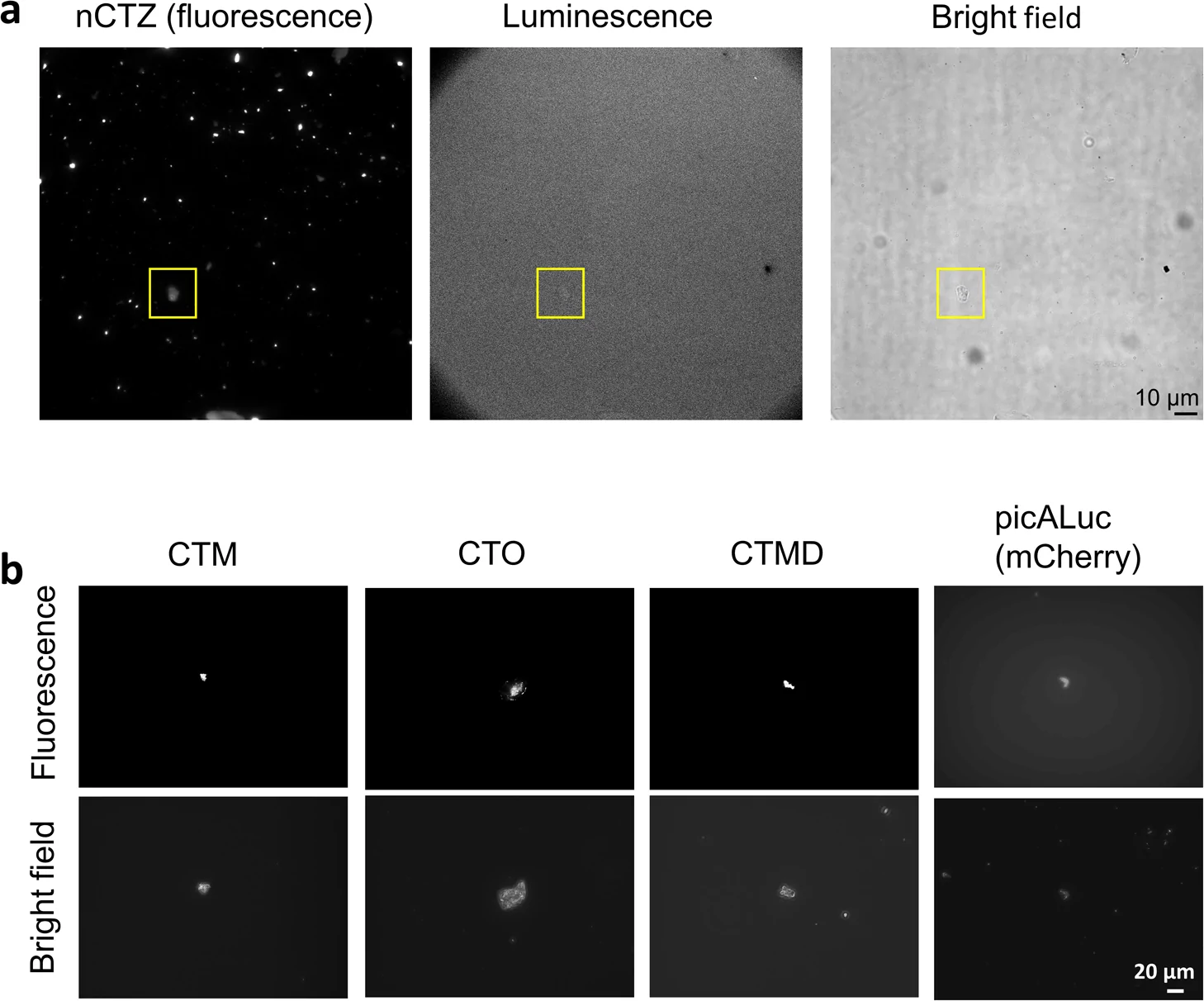
Colocalization of substrate, luminescence signal, substrate metabolites, and enzyme at large particles. (a) Colocalization of nCTZ and luminescence at large particles. A large particle where co-localization was observed is highlighted by a yellow square. (b) Colocalization of substrate metabolites (CTM, CTO, and CTMD) and enzyme at large particles.

Microscopic observation of a mixture of EtOH-diluted native CTZ (nCTZ) and picALuc (containing the surfactant NP-40) revealed that the mixture contained particles of various morphologies and sizes. The size of the large particles observed both in the fluorescence of the nCTZ signal and in the luminescence from the reaction of picALuc and nCTZ was approximately the same as that of the larger particles observed above (about 4 μm; Figure 5a). When this mixture was sonicated, the large particles almost disappeared; however, small particles were still observed. Because there was no change in the luminescence pattern after sonication (Figure S4), two possibilities were considered: i) the fluorescence signals of nCTZ and the luminescence signals were localized on the particles, and their accumulation in large particles reached a microscopically detectable level, or ii) particle formation was not associated with the change in luminescence patterns. The observation that surfactants with higher CMC require higher concentrations to sustain the luminescence signal supports the first possibility.

Each chemical species in the reaction—CTM (a non-luminescent metabolite of nCTZ), CTO (an oxidized form of nCTZ), and CTMD (a luminescent metabolite of nCTZ)—was also examined by fluorescence microscopy. Each species was dissolved in MeOH and mixed with NP-40, followed by similar microscopic observation. The results showed that the fluorescence signals of CTM, CTO, and CTMD were all co-localized with large particles (Figure 5b). Importantly, picALuc itself is a soluble protein under normal buffer conditions. In the presence of NP-40, however, micelles form above the CMC, creating hydrophobic microenvironments that preferentially partition hydrophobic molecules such as nCTZ and its reaction intermediates. Consistent with this, in a mixture of mCherry-fused picALuc, MeOH, and NP-40, the mCherry signal also appeared to colocalize with large particles. We interpret this apparent colocalization not as insolubilization or aggregation of picALuc, but as transient enrichment of the enzyme–substrate complex at the micelle surface, where the hydrophobic substrate and intermediates accumulate. These results collectively suggest that the highly hydrophobic nCTZ, CTM, CTO, and CTMD molecules are captured within or on the surface of the micelles, and that the luminescence reaction occurs preferentially in these micellar microenvironments.

### Reduction of product inhibition in luminescence by surfactants

**Figure 6:**
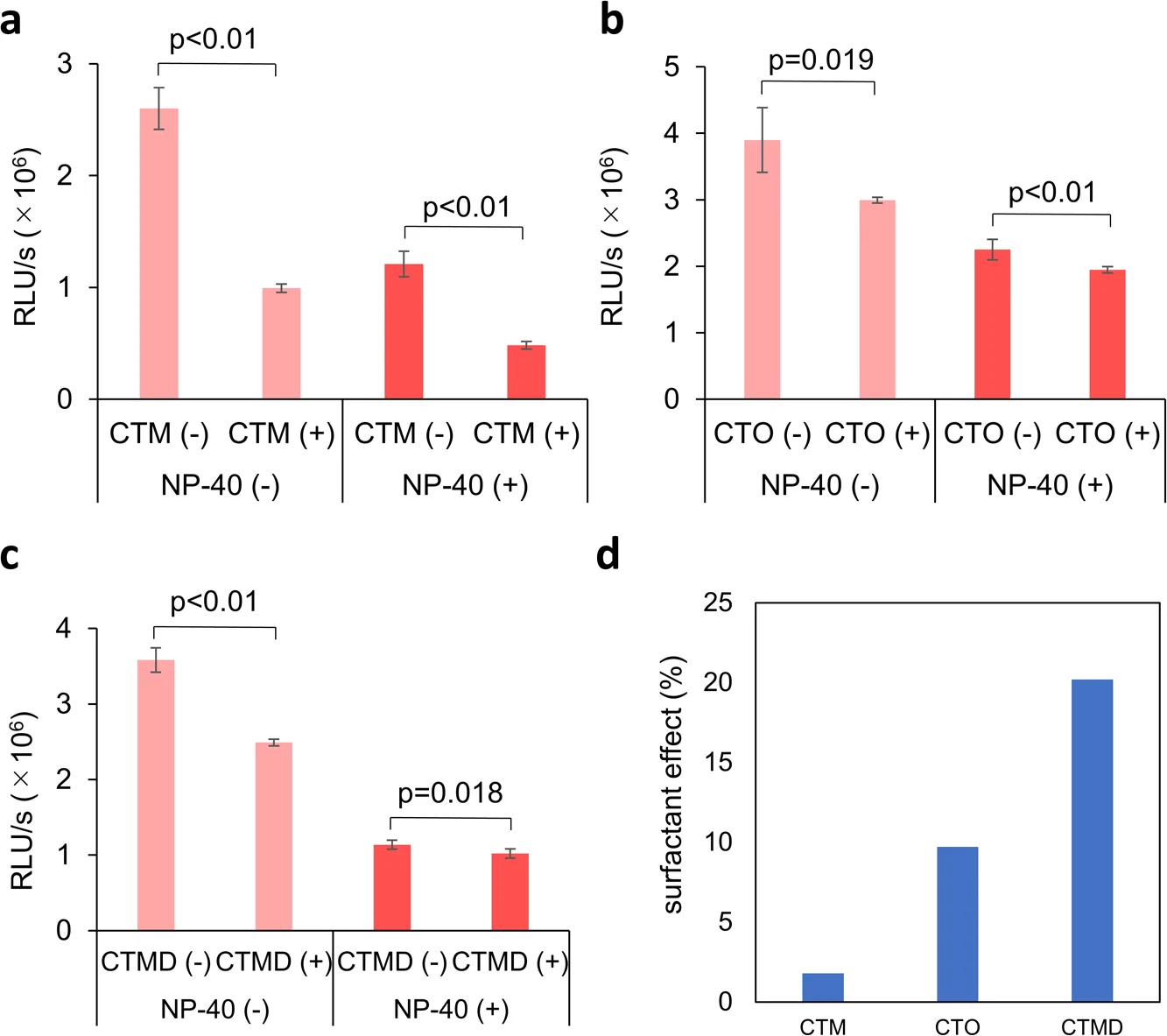
The luminescence inhibition of substrate metabolites and weakening of inhibition effect by surfactant addition. (a-c) The luminescence intensities treated with and without metabolites (CTM, CTO, and CTMD) and NP-40. Results are the mean±SD (n=3, t-test). (d) The decreased ratio of the luminescence intensities by surfactant addition.

The luminescent reactions of luciferases such as RLuc and GLuc are inhibited by substrate metabolites [28,29]. Based on the above results of the colocalization of enzymes, substrates, and metabolites, we investigated the possibility that increasing the amount of surfactant reduces the inhibitory effects of substrate metabolites, thereby sustaining luminescence. The luminescence intensity of the enzyme was measured after the addition of CTM, CTO, or CTMD to a mixture of picALuc and CTZ*h* in the presence or absence of NP-40. In the experiment, the initial luminescence intensities immediately after substrate addition were compared to avoid factors such as substrate consumption and oxidation, because the time to reach the maximum luminescence intensity differed depending on the conditions. As a result, in the absence of NP-40, luminescence inhibition was observed in all three chemical species (CTM, CTO and CTMD). In the absence of NP-40, a large decrease in the luminescence intensity due to CTM (62%) and a moderate decrease due to CTO or CTMD (23% and 30%, respectively) were observed, whereas in the presence of NP-40, the decrease due to CTO or CTMD was suppressed (13% and 10%, respectively), although a similar decrease was observed with CTM (60%, slightly suppressed) (Figures 6a–c). Regarding the surfactant effect, the differences in the reduction between the presence and absence of NP-40 in CTM, CTO, and CTMD are shown in Figure 6d. Although the addition of NP-40 had a minor effect on the CTM-induced luminescence inhibition (approximately 2%), it had an effect of approximately 10% on the CTO-induced luminescence inhibition, reaching approximately 20%. These results revealed that among the inhibitors of the luminescence reaction by chemical species, such as CTM, CTO, and CTMD, the inhibition by CTO and CTMD was alleviated by the addition of surfactants, which may lead to sustained luminescence.

### Production changes of metabolites by surfactants

**Figure 7:**
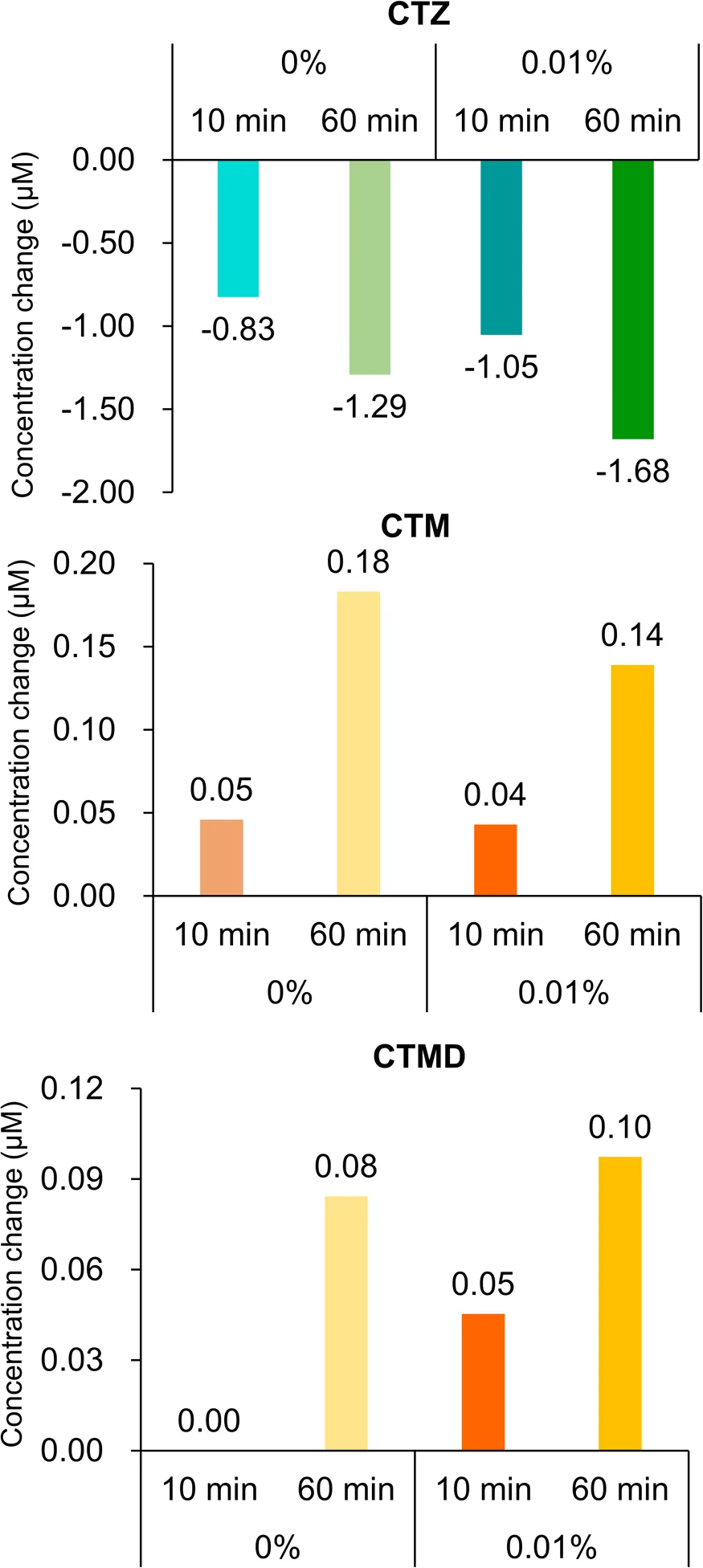
Changes of substrate consumption and metabolites productions by surfactant addition. The concentrations of CTZ, CTM, and CTMD with and without 0.01% NP-40 after reacting for 10 or 60 min at room temperature. Each value represents the mean of triplicate measurements.

To explore the consumption of nCTZ and the production of CTM, CTO, and CTMD [30] under surfactant conditions, the luminescence reaction was stopped at 10 and 60 min after adding MeOH-dissolved nCTZ to the picALuc dilution with or without NP-40. The amounts of nCTZ, CTM, CTO, and CTMD were measured using LC-MS (Figure S5). Note that the amounts of CTM, CTO, and CTMD before the luminescent reaction may be altered by the conversion of nCTZ to these compounds during storage. Therefore, the consumption of nCTZ and the production of CTM, CTO, and CTMD were calculated by subtracting the amounts present before the reaction. As shown in Figure 7, an increase in nCTZ consumption was observed after NP-40 treatment of approximately 30% for both 10 and 60 min. Moreover, with the addition of NP-40, the production of CTO remained almost unchanged (Figure S5), the production of CTM decreased by about 24% at 60 min, and the production of CTMD increased by approximately 15% at 60 min (Figure 7). These results indicate that the portion of the substrate converted to non-luminescent CTM decreased and that conversion to luminescence-related CTMD increased. Taken together, the luminescence intensity increased upon surfactant addition, likely due to enhanced nCTZ consumption during the short time period, whereas the extended luminescence duration was attributable to the preferential conversion of the substrate into the luminescence-related metabolite CTMD rather than the non-luminescent CTM over longer periods. In addition, considering the aforementioned microscopic observations, it appears that the luminescence-inhibitory chemical species were incorporated into the micelle-like particles, contributing to the persistence of luminescence.

## Discussion

In this study, we revealed that the intensity and duration of the luminescence phenomenon (the so-called L-L reaction) between the luciferase picALuc and the substrate CTZ can be increased by adding various surfactants, such as NP-40 and CHAPS. We demonstrated the usefulness of this effect (using NP-40 as an example) in a simplified ELISA system with biotinylated BSA and streptavidin-fused picALuc. One of the advantages of utilizing luciferase over commonly used chromogenic enzymes such as horseradish peroxidase [31,32] in general detection systems is its significantly wider dynamic range. However, the disadvantage is that the signal decays very quickly, making it difficult to measure without specialized equipment, such as a luminometer. By utilizing the effect of the surfactant to extend the luminescence time in this study, it is expected that the aforementioned disadvantages will be eliminated and the applications of the detection system will be further expanded. From another perspective, surfactants with higher CMC require solutions with higher concentrations to sustain luminescence. Regarding the effects on luminescence intensity and time, the relationships between the CMC values and effective concentrations have not yet been quantitatively clarified. In addition, the differences in surfactant properties, such as ionic, non-ionic, and amphoteric properties, which affect these effects remain unknown. Further investigations are required to determine the effective concentrations and properties of surfactants for enzyme function.

In the L-L reaction involving CTZ (luciferin), the excited-state amide anions of CTMD (oxyluciferin) and CO_2_ are generated via the high-energy intermediate dioxetanone. When the excited-state amide anion of the CTMD relaxes to the ground state, light emission occurs. CTMD form different light emitters, such as the neutral form (386–423 nm), phenolate anion (480–490 nm), amide anion (465–479 nm), and pyrazine anion (530–565 nm) [33]. However, many unknowns remain regarding other metabolites (reaction products) such as non-luminescent CTM and CTO, which undergo different reactions. In this study, we revealed that all three metabolites (CTMD, CTM, and CTO) inhibited luminescence (i.e., luminescence intensity decreased) and that the addition of surfactants alleviated this inhibition of luminescence intensity. Furthermore, DLS experiments to investigate the cause revealed that micelle-like particles were formed by the surfactant (NP-40) and alcohol (EtOH or MeOH), and microscopic experiments revealed that the luminescence of the substrate (nCTZ) and the fluorescence of the metabolites (CTM, CTO, and CTMD) were concentrated on these micelle-like particles. Taken together, it is understood that the addition of surfactant at a concentration related to the CMC causes the formation of micelle-like particles; the L-L reaction occurs in the vicinity, and the removal of the inhibitors enhances the luminescence intensity and elongates the luminescence time.

Luminescent bacteria are widely used in soil and water pollution detection [34,35] and toxicity tests [36], in which the lux operon is activated and functions as a luminescent reporter (or biosensor). Moreover, artificial luciferases TurboLuc and NanoLuc are thought to be effective for high-throughput screening because of their small size [11,37]. When performing measurements, it is necessary to consider the presence of surfactants and the effects of chemicals that create hydrophobic particles. The effect of improving enzymatic reactions by adding surfactants obtained here may have limitations that cannot be directly applied to cells and tissues; however, it will be useful in the field of biological science that uses measurements in test tubes, such as sensing reactive oxygen species and enzymes, protein–protein interaction assays, and high-throughput screening [38].

## Conclusion

In this study, we investigated the luminescence properties of luciferase picALuc after the addition of various surfactants. The enhancement of luminescence intensity and extension of luminescence time were observed, and these effects were verified using a simplified ELISA system. Furthermore, DLS measurements confirmed that micelle-like particles were formed only by the surfactant and alcohol (MeOH or EtOH), and microscopic observations revealed that both the luminescence due to the L-L reaction and the fluorescence of its metabolites CTM, CTO, and CTMD were located near the particles. It was also found that, although all metabolites, CTM, CTO, and CTMD, inhibited luminescence, the addition of surfactants specifically alleviated the inhibition of CTO and CTMD. The luminescence properties may be altered by suppressing the production of nonluminescent CTM and incorporating the luminescent product CTMD into micelle-like particles. The findings of this study provide important insights into the luminescence mechanism of luciferase and open new doors for the possibility of surfactant addition to enzymatic reactions.

## Conflict of interest

Y. O. M., K. M., and R. T. are employees of Shimadzu Corporation. T. F., G. K., N. K., S. A. M., M. H., and T. N. received research funds from Shimadzu Corporation.

## Author contributions

Y. O. M. and T. F. conceived the concept and prepared the manuscript. K.M. performed LC-MS measurements. G. K., N. K., and S. A. M. synthesized CTO, CTM, and CTMD, respectively. M. H. and T. N. performed microscopic observations. R. T. supported the analysis of the micelle-like particles. Y. O. M. performed all other experiments.

## Data availability

The datasets generated and/or analyzed in the current study are available from the corresponding author upon reasonable request.
