# Supplementary material for: Effects of surfactants on luminescence intensity and duration of the minimal luciferase picALuc
Source: Biophys Physicobiol. 2026 Jul 11;23(3):e230023. doi: 10.2142/biophysico.bppb-v23.0023 (PMC13542556; doi:10.2142/biophysico.bppb-v23.0023)
Supplement: Supplementary file 1 [file 23_e230023_1.pdf]

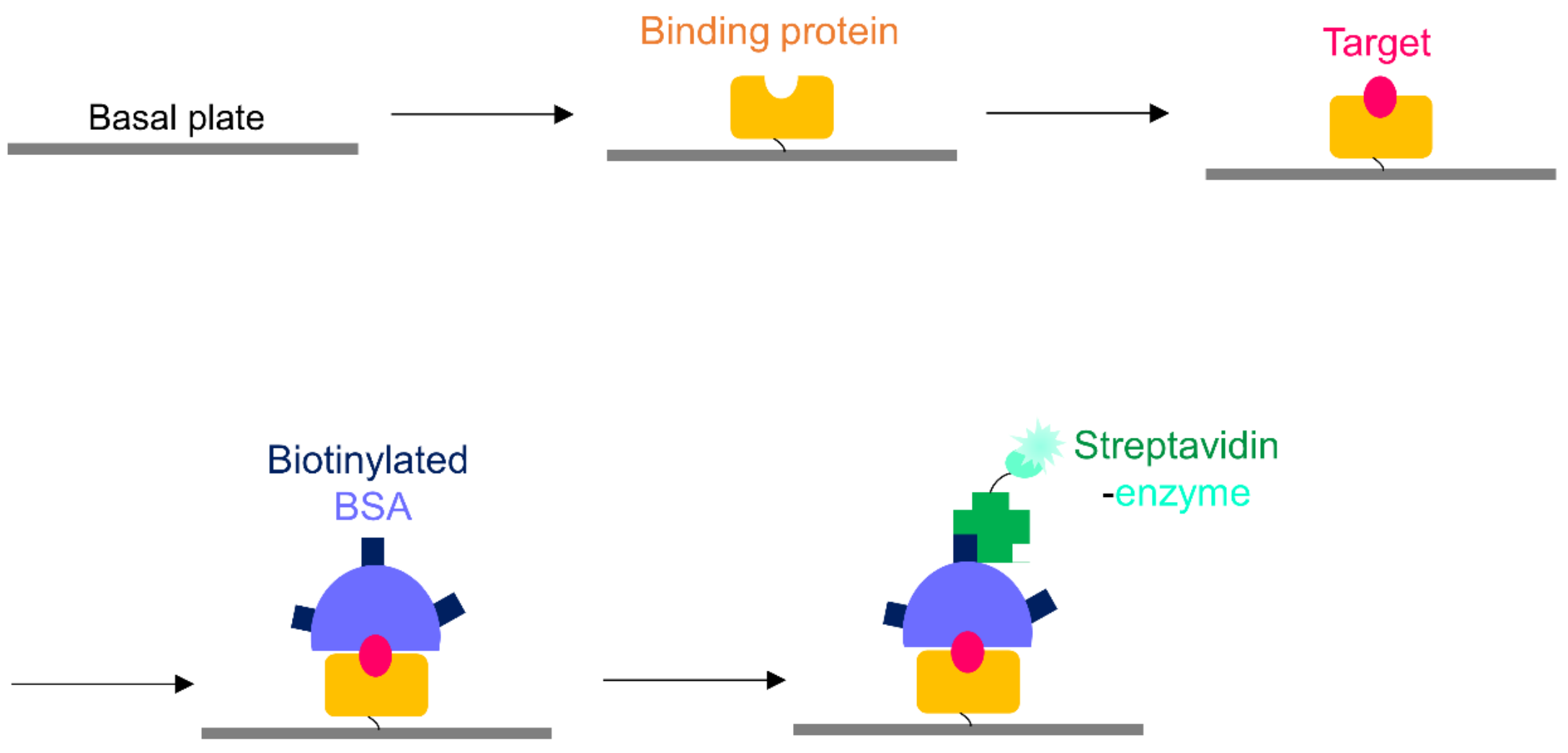

Figure S1. Schematic diagram of the common detection system using biotin–streptavidin.

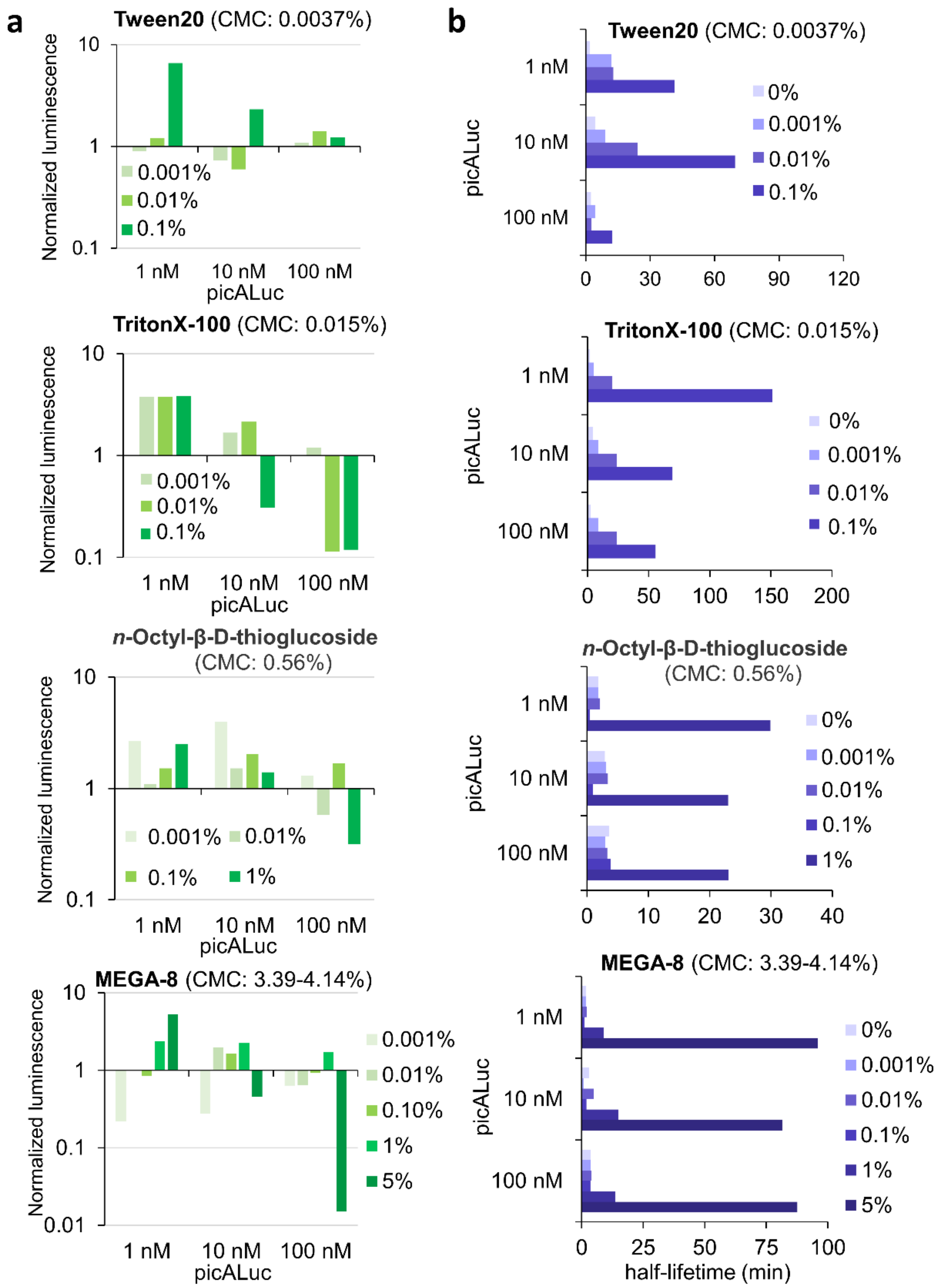

Figure S2. Changes of luminescence intensities (a) and decays (b) of picALuc by adding surfactants.

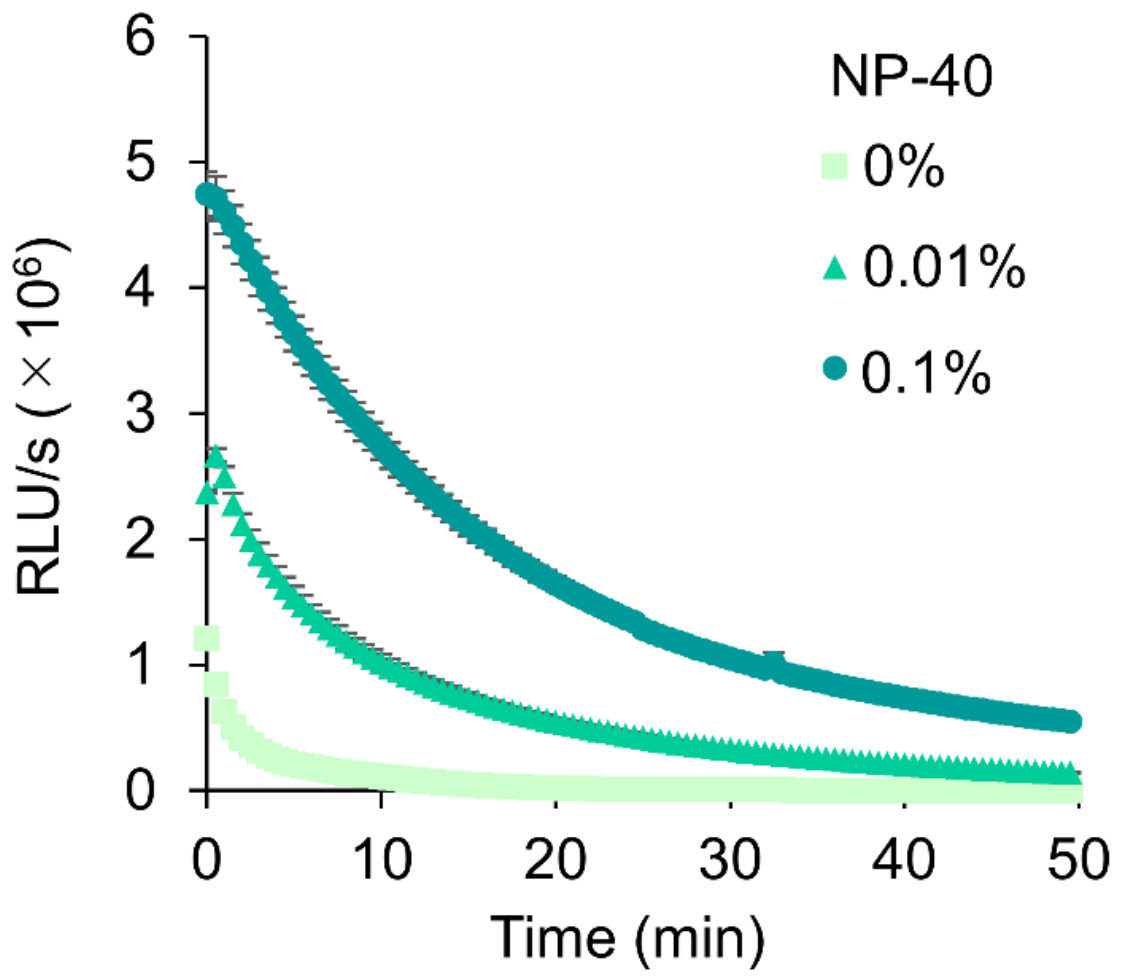

Figure S3. Changes of luminescence patterns of picALuc by adding surfactant dissolved in MeOH. nCTZ (40 μM) dissolved with MeOH was reacted with picALuc (50 nM) in PBS containing 0.01% NP-40. Results are the mean ± SD (n = 3).

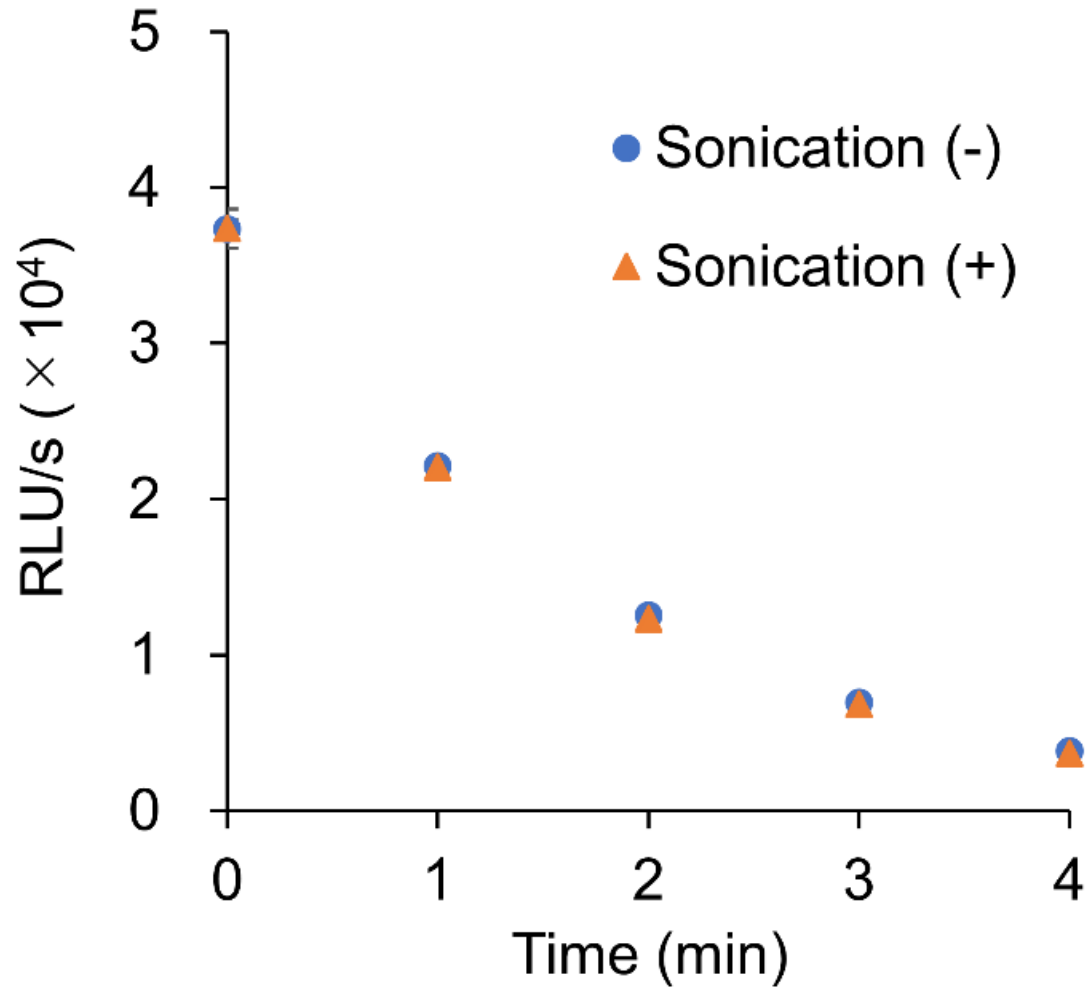

Figure S4. Retention of luminescence intensity when larger particles were broken by sonication.

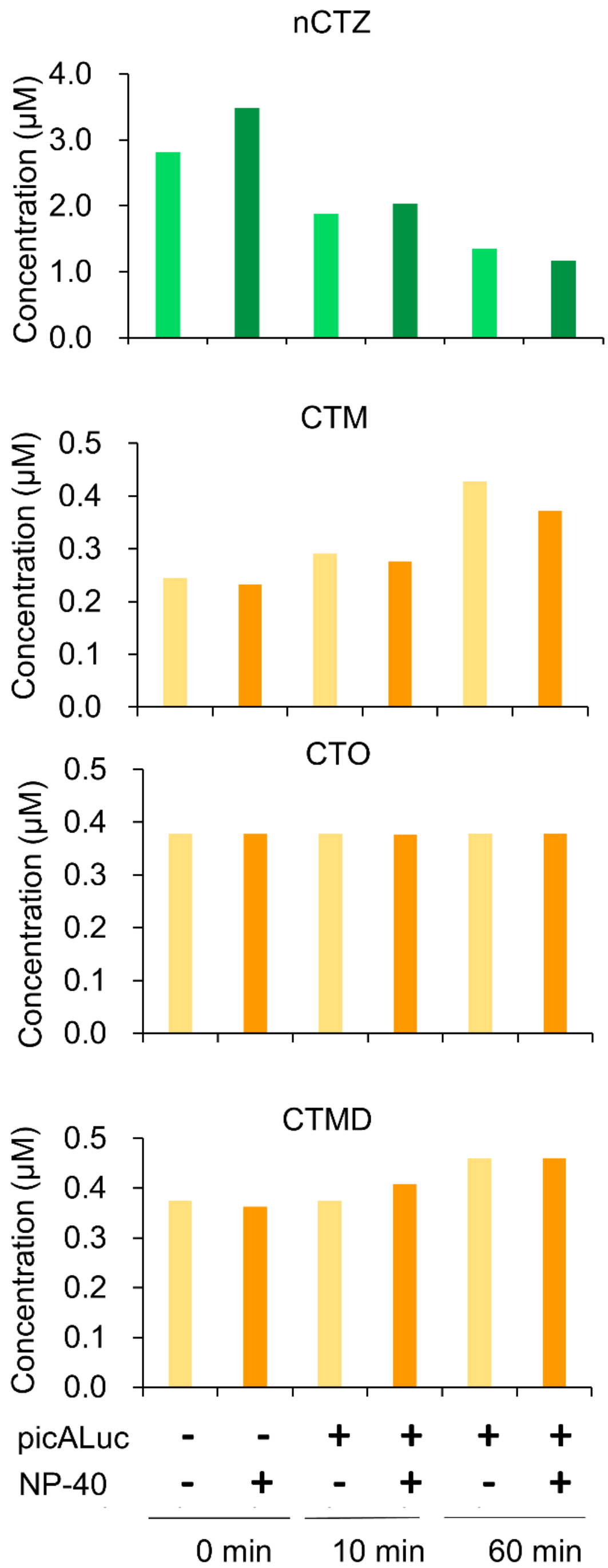

Figure S5. Measurement of substrate consumption and metabolites production with and without surfactant.

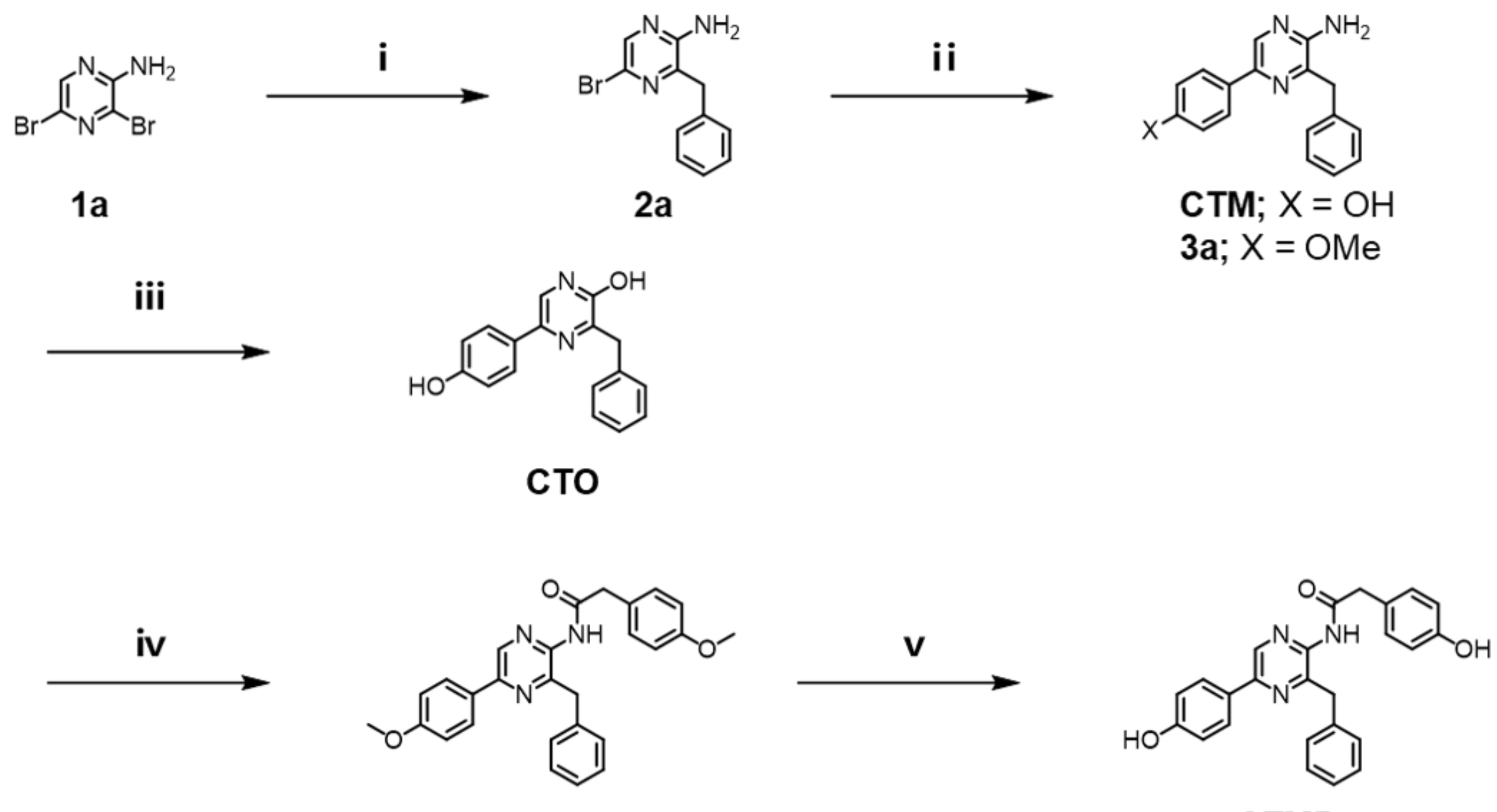

Scheme S1. Synthetic scheme for CTZ compounds (**CTM, CTO,** and **CTMD**).

Compounds **2a** was synthesized based on previously reported procedures (Kamiya et al. *Int. J. Mol. Sci.* **2022**, *23*(21), 13047).

i; Zinc chloride, Benzylmagnesium chloride, Bis(triphenylphosphine)palladium(II) Dichloride, tetrahydrofuran, room temperature. ii; Tetrakis(triphenylphosphine)palladium(0), boronic acid, 1,4-dioxane, 1 M Na_2CO_3 aq, 110 °C. iii; Sodium nitrite, concentrated sulfuric acid, 0°C to 40 °C. iv; 4-methoxyphenylacetyl chloride, pyridine, 0 °C to room temperature. v; Boron tribromide, dichloromethane, -20 °C to room temperature.

Method S1. Specific procedures for the organic synthesis and the corresponding NMR and mass data of **CTM**, **3a**, **CTO**, **4a**, and **CTMD**.

Coelenteramine (**CTM**): 4-(5-amino-6-benzylpyrazin-2-yl)phenol

Compound **2a** (927 mg, 3.66 mmol) and 4-Hydroxyphenyl boronic Acid (909 mg, 6.59 mmol) were dissolved in 1,4-dioxane (15 mL) at room temperature. Tetrakis(triphenylphosphine)palladium(0) (211 mg, 0.18 mmol) and 1M Na_2CO_3 aq (10 mL) were added to the mixture for 2.5 hours at 110 ℃ under argon. After the reaction was completed, the product was extracted with ethyl acetate three times. The organic phase was dried over Na_2SO_4 and evaporated. The residue was separated by column chromatography (hexane/ethyl acetate = 1/1) to obtain **Coelenteramine (CTM)** (827 mg, 2.98 mmol, 45 %, yellow solid).

^{1}H-NMR (500 MHz, acetone-d_6) δ 8.34 (s, 1H), 7.84 (dd, J = 11.5, 2.9 Hz, 2H), 7.36 (d, J = 7.4 Hz, 2H), 7.31-7.28 (m, 2H), 7.21 (t, J = 7.2 Hz, 1H), 6.90 (dd, J = 11.5, 2.9 Hz, 2H), 5.44 (s, 2H), 4.16 (s, 2H).

^{13}C-NMR (126 MHz, acetone-d_6) δ 158.3, 152.9, 142.1, 140.7, 139.0, 137.1, 130.2, 129.7, 129.3, 127.5, 127.2, 116.3, 40.6

HR-MALDI-MS: m/z: [M+H]+ calcd for $C_{17}H_{16}N_3O$, 278.11944; found, 278.12944.

HPLC purity: 98.3% (gradient elution, CH_3CN/0.1% formic acid in H_2O = 10:90 to 90:10 over 20 min, UV 254 nm, Rt = 9.46 min).

Compound **3a**: 3-benzyl-5-(4-methoxyphenyl)pyrazin-2-amine

Compound **2a** (996 mg, 3.79 mmol) and 4-Methoxyphenyl boronic Acid (691 mg, 4.54 mmol) were dissolved in 1,4-dioxane (15 mL) at room temperature. Tetrakis(triphenylphosphine)palladium(0) (281 mg, 0.19 mmol) and 1M Na_2CO_3 aq (15 mL) were added to the mixture for 1 hours at 110 °C under argon. After the reaction was completed, the product was extracted with ethyl acetate three times. The organic phase was dried over Na_2SO_4 and evaporated. The residue was separated by column chromatography (hexane/ethyl acetate = 2/1 to 1/1) to obtain the compound **3a** (971 mg, 3.33 mmol, 73 %, yellow solid).

^{1}H-NMR (500 MHz, acetone-*d₆*) δ 8.14 (s, 1H), 7.70 (dt, J = 9.4, 2.3 Hz, 2H), 7.14 (d, J = 7.4 Hz, 2H), 7.07 (t, J = 7.7 Hz, 2H), 6.99 (t, J = 7.4 Hz, 1H), 6.76 (td, J = 6.0, 3.4 Hz, 2H), 5.27 (s, 2H), 3.94 (s, 2H), 3.61 (s, 3H), 2.58 (s, 1H)

^{13}C-NMR (126 MHz, acetone-d_6) δ 160.5, 153.0, 141.6, 140.8, 138.9, 137.3, 131.1, 129.8, 129.3, 127.3, 127.2, 114.8, 55.6, 40.5

HR-MALDI-MS: m/z: [M+H]+ calcd for $C_{18}H_{18}N_3O$, 292.14444; found, 292.14503.

CTO: 3-benzyl-5-(4-hydroxyphenyl)pyrazin-2-ol

Sodium nitrite(36 mg, 0.52 mmol) was dissolved in concentrated sulfuric acid (2 mL) at 0 °C and stirred at 60 °C for 1 hour. The mixture was cooled at 0 °C. **CTM** was added to the mixture at 0 °C and stirred at 40 °C for 1 hour. After cooling to room temperature, the mixture was poured onto crushed ice. The mixture was extracted with ethyl acetate three times. The organic phase was dried over Na_2SO_4 and evaporated. The residue was separated by automated flash chromatography (Smart Flash EPCLC AI-580S, Universal Columns, chloroform /methanol = 99/1 to 9/91) to obtain **CTO** (23 mg, 0.083 mmol, 19 %, pale yellow solid).

^{1}H-NMR (500 MHz, acetone-d_6) δ 7.71 (dt, J = 9.2, 2.4 Hz, 2H), 7.68 (s, 1H), 7.42 (d, J = 6.9 Hz, 2H), 7.29-7.26 (m, 2H), 7.18 (t, J = 7.4 Hz, 1H), 6.87 (dt, J = 9.2, 2.4 Hz, 2H), 4.12 (s, 2H)

^{13}C-NMR (126 MHz, acetone-d_6) δ 158.2, 158.0, 155.6, 139.1, 133.4, 130.2, 129.0, 128.8, 127.0, 126.9, 121.2, 116.2, 40.0

HR-MALDI-MS: m/z: [M+H]+ calcd for $C_{17}H_{15}N_2O_2$, 279.11280; found, 279.11362.

HPLC purity: 96.8% (gradient elution, CH_3CN/0.1% formic acid in H_2O = 10:90 to 90:10 over 20 min, UV 254 nm, Rt = 9.23 min).

Compound **4a**: *N*-(3-benzyl-5-(4-methoxyphenyl)pyrazin-2-yl)-2-(4-methoxyphenyl)acetamide

Compound **3a** (777 mg, 2.67 mmol) was dissolved in pyridine (30 mL) at 0 °C under argon. 4-methoxyphenylacetyl chloride (0.53 mL, 3.47 mmol) was added to the mixture at 0 °C to room temperature for 12 hours. After the reaction was completed, the product was extracted with chloroform and 1M HCl aq three times. The organic phase was dried over Na_2SO_4 and evaporated. The residue was separated by column chromatography (hexane/ethyl acetate = 4/1 to 2/1) to obtain the compound **4a** (797 mg, 1.81 mmol, 68 %, pale yellow solid).

^{1}H-NMR (500 MHz, acetone-*d₆*) δ 8.76 (s, 1H), 8.06 (td, J = 6.0, 3.4 Hz, 2H), 7.34 (d, J = 8.6 Hz, 2H), 7.23-7.19 (m, 2H), 7.17-7.14 (m, 1H), 7.11 (d, J = 6.9 Hz, 2H), 7.05 (d, J = 8.6 Hz, 2H), 6.92 (td, J = 6.0, 3.4 Hz, 2H), 4.16 (s, 2H), 3.87 (s, 3H), 3.80 (s, 3H), 3.71 (s, 2H)

^{13}C-NMR (126 MHz, acetone-d_6) δ 170.9, 162.0, 159.8, 149.7, 144.7, 139.5, 137.7, 131.3, 130.0, 129.5, 129.1, 128.9, 128.2, 127.0, 115.1, 114.8, 55.7, 55.5, 43.0, 41.3

HR-MALDI-MS: m/z: [M+H]+ calcd for $C_{27}H_{26}N_3O_3$, 440.19687; found, 440.19569.

Coelenteramide (**CTMD**): *N*-(3-benzyl-5-(4-hydroxyphenyl)pyrazin-2-yl)-2-(4-hydroxyphenyl)acetamide

Compound **4a** (300 mg, 0.68 mmol) was dissolved in dehydrated dichloromethane (20 mL) and stirred at -20 °C under argon. Boron tribromide (17% in dichloromethane, 1 moL/L) (3 mL, 0.31 mmol) was added to the mixture at -20 °C, and the mixture was stirred for 12 hours at room temperature under argon. After the reaction was completed, saturated $NaHCO_3$ aq was added at 0 °C, and the product was extracted with chloroform one time and ethyl acetate three times. The organic phase was dried over Na_2SO_4 and evaporated. The residue was separated by column chromatography (hexane / ethyl acetate = 3/1 to 1/1) to obtain **CTMD** (221 mg, 0.54 mmol, 79%, pale yellow solid).

^{1}H-NMR (500 MHz, acetone-d_6) δ 8.72 (s, 1H), 7.98 (d, J = 9.2 Hz, 2H), 7.25 (d, J = 8.0 Hz, 2H), 7.21 (t, J = 7.4 Hz, 2H), 7.15 (t, J = 7.4 Hz, 1H), 7.11 (d, J = 8.0 Hz, 2H), 6.95 (d, J = 8.6 Hz, 2H), 6.83 (d, J = 8.6 Hz, 2H), 4.15 (s, 2H), 3.67 (s, 2H)

^{13}C-NMR (126 MHz, acetone-d_6) δ 171.0, 159.9, 157.4, 150.0, 144.5, 139.5, 137.5, 137.5, 131.3, 130.0, 129.0, 128.5, 127.1, 127.0, 116.6, 116.2, 43.1, 41.3

HR-MALDI-MS: m/z: [M+H]+ calcd for $C_{25}H_{22}N_3O_3$, 412.16557; found, 412.16537.

HPLC purity: 92.4% (gradient elution, CH_3CN/0.1% formic acid in H_2O = 10:90 to 90:10 over 20 min, UV 254 nm, Rt = 10.24 min).
